# The Role of Cholesterol at the CHOL2 Site and the Dimeric Interface in the Regulation of Serotonin Transporter Function and Dimerization

**DOI:** 10.3390/biom16030472

**Published:** 2026-03-21

**Authors:** Jian Yang, Chan Li, Xingyu Huang, Yuan-Wei Zhang

**Affiliations:** School of Life Sciences, Guangzhou University, Guangzhou 510006, China; 2112314035@e.gzhu.edu.cn (J.Y.); lichan@gzhu.edu.cn (C.L.); 1112214006@e.gzhu.edu.cn (X.H.)

**Keywords:** serotonin transporter, cholesterol, regulation, transport function, conformation, dimerization

## Abstract

Serotonin transporter (SERT) precisely regulates serotonin (5–HT) signaling in the central nervous system and is a major target of antidepressants for the treatment of major depressive disorder. Despite significant progress in characterizing its structure and transport mechanism, the regulation of SERT function by various modulators remains to be fully understood. In the present study, we focused on two potential cholesterol sites in human SERT to investigate cholesterol occupation at these sites and its functional relevance by biochemical approaches. Mutations of an intramolecular site (CHOL2) significantly decreased both specific transport activity and *K_m_* for 5–HT and stabilized the transporter in an inward-facing conformation. In addition, our NanoBiT luminescent assay for protein–protein proximity demonstrated that cholesterol mediated the protomer–protomer interactions by residing in a site at the dimeric interface. Mutations of the interfacial site remarkably reduced the interactions between SERT protomers and substantially impaired their transport activity. The structural analysis indicated that the residues participating in cholesterol residing in the interfacial site were conformationally sensitive. Thus, we have proposed that cholesterol at these sites could play a vital role in the regulation of SERT function by a conformational mechanism. Our study has provided new insights into the molecular mechanism by which cholesterol can regulate SERT function and dimerization.

## 1. Introduction

Serotonin transporter (SERT) accurately modulates serotonin (5–hydroxytryptamine, 5–HT) signaling in the central nervous system (CNS) by the reuptake of 5–HT from the synaptic cleft after its release from presynaptic neurons. Dysfunction of 5–HT signaling has been demonstrated to be associated with the pathophysiology of many psychiatric disorders [1,2,3]. It is worth noting that SERT is a major target of drug abuse, such as cocaine and amphetamines, as well as selective serotonin reuptake inhibitors (SSRIs), such as fluoxetine, citalopram, and imipramine, which are commonly used for the treatment of major depressive disorder [4,5,6].

SERT is a member of the neurotransmitter sodium symporter superfamily (NSS) and shares a common structural feature and similar transport mechanism with other NSS transporters [7,8]. Together with transporters for dopamine (DAT) and norepinephrine (NET), SERT also belongs to the monoamine transporter (MAT) subfamily. Because of its importance in 5–HT neurotransmission, illustrating the structure and transport mechanism of SERT has been a main research focus during the past few decades. Thus, many high-resolution structures of SERT bound with ligands, ions, and substrate in several conformational states have been resolved, providing a structural basis to understand its transport or inhibition mechanisms [9,10]. Increasing evidence has indicated that SERT transports 5–HT across the plasma membrane by an alternating access mechanism, by which the central binding site is alternately exposed to the extracellular or cytoplasmic medium for substrate binding or release by conformational changes that open and close the substrate permeation pathway [11,12,13]. Despite significant progress in characterizing its structure and transport mechanism, the regulation of SERT by various modulators, such as membrane components, post-translational modifications, signal transduction pathways, and so on, remains to be fully understood [14,15,16]. One challenge has been to explore the regulatory mechanism of SERT by cholesterol.

Cholesterol is enriched in the plasma membrane in the brain and plays an important role in the brain development and neuronal function [17,18]. Early studies have indicated that the depletion of membrane cholesterol decreases the ligand binding and substrate transport of SERT [19,20]. Similar observations have also been seen with DAT [21,22]. Following this, a bound cholesterol molecule has been observed at a site, designated as CHOL1, formed by transmembrane domains (TMs) 1, 5, and 7 in the structures of *Drosophila melanogaster* DAT (dDAT) [23,24] and human SERT (hSERT) [9]. The following mutagenesis analysis for the CHOL1 residues have shown that the mutations affect the interaction between SERT and cholesterol, which modulates substrate affinity, transport velocity, and turnover rates by manipulating SERT conformation [25].

In addition to the CHOL1 site, another cholesterol site, named CHOL2, was first observed in the structures of dDAT [23]. The recent structures of SERT from porcine brain (pSERT) also revealed two molecules of a cholesterol analog, cholesteryl hemisuccinate (CHS), that can occupy the CHOL1 and CHOL2 sites [26]. The CHOL2 site is located in a groove formed by TMs 2, 7, and 11 in dDAT and pSERT, but no information is available for the CHOL2 site in hSERT. In addition, the recently resolved structures of a NET dimer have defined a dimeric interface that is mediated predominantly by cholesterol and lipid molecules [27]; however, cholesterol-mediated SERT dimerization and its functional consequence have not been reported.

To better understand the regulatory mechanism of SERT by cholesterol, there are still several questions to be answered: (1) Does a cholesterol molecule occupies at the corresponding CHOL2 site in hSERT? (2) What is the role of a cholesterol molecule in the CHOL2 site in the regulation of SERT? (3) Does SERT forms a cholesterol-mediated dimer? (4) Do cholesterol-mediated dimeric interactions affect SERT activity? In the present study, we focus on the CHOL2 site and the dimeric interface of SERT to explore the role of cholesterol in the regulation of SERT by using biochemical approaches. Our study provides new insights into the molecular mechanism by which cholesterol regulates SERT function and dimerization.

## 2. Materials and Methods

### 2.1. Materials

HEK 293T and HeLa cells were obtained from the American Type Culture Collection. [^3^H]5–HT (27.1 Ci/mmol) and [^3^H]imipramine (53.5 Ci/mmol) were purchased from PerkinElmer Life Sciences (Shelton, CT, USA). 5–HT and sulfo–NHS–biotin were obtained from Apexbio (Houston, TX, USA). Streptavidin agarose gel and Super Signal West Pico were from ThermoFisher Scientific (Waltham, MA, USA). 2–Aminoethyl methanethiosulfonate hydrobromide (MTSEA) was purchased from GlpBio (Monterey, CA, USA). All other reagents were of an analytical grade.

### 2.2. Mutagenesis and Stable Cell Line Generation

The lentiviral plasmids (Lenti–EF–1α–SERT–BSD or Lenti–EF–1α–S277C/X5C–BSD) encoding C-terminal FLAG-tagged SERT were used as templates for mutagenesis, as described previously [28]. All mutants were generated using the Mut Express II Fast Mutagenesis Kit (Vazyme, Nanjing, China) and confirmed by full-length DNA sequencing.

HEK 293T or HeLa cells were maintained with 5% CO_2_ at 37 °C in the Dulbecco’s modified Eagle’s medium, supplemented with 10% of fetal bovine serum (Newzerum, Christchurch, New Zealand), streptomycin (100 μg/mL), and penicillin (100 U/mL). Stable cell lines were generated according to the protocol described previously in reference [29]. Lentivirus was prepared by transfecting HEK 293T cells with a mixture of a lentiviral plasmid and two other packaging vectors, psPAX2 and pMD2G. HEK 293T or HeLa cells were then infected by lentivirus with polybrene and selected by blasticidin S at a concentration of 10 μg/mL. The culture medium was changed every 3 days until colonies of blasticidin S-resistant cells were formed.

### 2.3. Cell Membrane Preparation

HEK 293T or HeLa cells expressing SERT or its mutants were harvested into 25 mM Hepes buffer, pH 7.4, containing 120 mM NaCl, 5 mM KCl, 2.2 mM MgSO_4_, 1.2 mM CaCl_2_, 0.5% of a protease inhibitor mixture and 100 μM of phenylmethylsulfonyl fluoride, and then disrupted by three cycles of freeze–thawing. The resulting crude membrane fraction was collected via centrifugation at 15,000× *g* for 20 min at 4 °C and stored at –80 °C until use.

### 2.4. Cholesterol Depletion and Replenishment

The depletion of membrane cholesterol was performed by incubating the cells or crude membranes with methyl-β-cyclodextrin (MβCD) (Macklin, Shanghai, China) at various concentrations (0 to 20 mg/mL) in KRH buffer (20 mM Hepes, pH 7.4, 120 mM NaCl, 1.3 mM KCl, 2.2 mM CaCl_2_, 1.2 mM MgSO_4_, and 0.1% glucose) for 30 min at 22 °C with gentle shaking, as described previously in reference [30]. The cells or cell membranes were then used for transport or ligand binding assays. MβCD-mediated cholesterol replenishment was performed according to the protocol reported previously in references [22,30]. A mixture of MβCD and cholesterol (Macklin, Shanghai, China) at a molar ratio of 12.5:1 in KRH buffer was obtained by vortexing, sonicating, and rotating overnight. The next day, cholesterol-depleted cell membranes were incubated with the MβCD–cholesterol mixture at various concentrations at 22 °C for 2 h and the membranes were then collected by centrifugation at 15,000× *g* at 4 °C for 15 min for further use. The cholesterol contents in the cells or cell membranes were measured using the Amplex red cholesterol assay kit (Beyotime, Shanghai, China).

### 2.5. 5–HT Uptake or Imipramine Binding Assay

[^3^H]5–HT uptake was assayed with HEK 293T or HeLa cells expressing SERT or its mutants at 22 °C, as described previously in reference [28]. In brief, 5–HT uptake was initiated by incubating HEK 293T or HeLa cells grown in 96-well poly–*D*–lysine-precoated plates with [^3^H]5–HT at a final concentration of 20 nM, and the incubation was then continued for 10 min. The assays were terminated by three rapid washes with ice–cold phosphate-buffered saline (PBS buffer, 137 mM NaCl, 2.7 mM KCl, 4.3 mM Na_2_HPO_4_, and 1.4 mM KH_2_PO_4_, pH 7.4). The cells were then solubilized in 30 µL of 0.1 M NaOH for 30 min. The extent of [^3^H]5–HT accumulated in the cells was determined by liquid scintillation spectrometry with a PerkinElmer Microbeta2 plate counter (Shelton, CT, USA). For kinetic analysis, the cells were incubated with 20 nM [^3^H]5–HT together with unlabeled 5–HT at a range of total 5–HT concentrations from 0.02 to 20 µM. All transport measurements were corrected by subtracting a non-specific transport measured in the presence of 100 µM fluoxetine. The protein concentration was determined with the enhanced BCA protein assay kit (Beyotime, Shanghai, China).

[^3^H]Imipramine binding assay for crude membranes was performed in 96-well filtration plates, according to previous descriptions in reference [31]. Cell membranes were added to each well and washed twice by filtration with KRH buffer. [^3^H]Imipramine binding was initiated by the addition of 100 μL of KRH buffer containing 2 nM of [^3^H]imipramine. The binding was allowed to proceed at 22 °C for 1.5 h with gentle rocking. The reaction was stopped by washing three times with ice-cold KRH buffer. The filters were removed from the plates and counted.

### 2.6. Cell Surface Biotinylation

Cell surface expression of SERT WT or its mutants was determined using the membrane-impermeant biotinylation reagent sulfo–NHS–biotin [32,33]. Briefly, cells expressing SERT or its mutants were treated with sulfo–NHS–biotin on ice in a biotinylation buffer (20 mM Hepes, pH 8.5, 2 mM CaCl_2_, 150 mM NaCl) for 30 min, and then rinsed with 100 mM of glycine in PBS for 20 min on ice to quench the excess sulfo–NHS–biotin. The cells were lyzed and biotinylated proteins were recovered by incubating the cell lysates with streptavidin agarose overnight at 4 °C. The biotinylated proteins were then eluted into 100 µL of SDS–PAGE sample buffer and separated on 10% SDS–polyacrylamide gels. SERT was visualized by a Western blot with monoclonal anti-FLAG antibodies (1:1000) against a FLAG epitope tag at the C-terminus of SERT. SERT cell surface expression was determined by quantitative luminescence imaging using an eBlot touch imager (Shanghai, China).

### 2.7. Cytoplasmatic Cysteine Accessibility Measurement

Cysteine accessibility in the cytoplasmic pathway was measured by incubating crude cell membranes prepared from cells stably expressing S277C/X5C or its mutants with MTSEA at various concentrations (0–1 mM) in a binding buffer (25 mM Hepes, pH 7.4 and 150 mM NMDGCl) for 15 min at 22 °C, as described previously in reference [34]. After washing three times with 100 μL of the binding buffer to remove unreacted MTSEA, the membranes were incubated with 2 nM of [^3^H]imipramine in KRH buffer at 22 °C for an additional 1.5 h, followed by washing three times with ice-cold PBS. Imipramine binding to the membranes on the fiber glass filters in 96-well filtration plates was measured by liquid scintillation spectrometry.

### 2.8. NanoBiT Luciferase Luminescent Assay

The LgBiT (17.6 kDa) or SmBiT (1.3 kDa) of the NanoBiT system (Promega, Madison, WI, USA) were fused to the N-terminus of SERT or its mutants, according to the manufacturer’s instruction. The cells in 12-well plates were transiently co-transfected with plasmids encoding LgBiT–SERT and SmBiT–SERT, with a cDNA amount ratio of 1:1 (400 ng/400 ng). Twenty-four hours after transfection, the cells were reseeded into a 96-well plate and grown to 100% confluence. The luciferase substrate was then added, and luminescent signals were immediately measured by the Infinite M–Plex microplate reader (Tecan, Männedorf, Switzerland). To obtain a NanoBiT signal curve, the cells were transiently co-transfected with a plasmid encoding LgBiT–SERT at a constant amount (400 ng) and a plasmid encoding SmBiT–SERT at a gradually increasing amount (0–400 ng), and the luminescent signals were then measured.

### 2.9. Statistical Analysis

All data were derived from experiments replicated at least three times with triplicate measurements each time. Values are expressed as mean ± SEM. Asterisks indicate significance at a *p* < 0.05. All statistical analyses were performed using one-way ANOVA, followed by Bonferroni’s multiple comparisons test.

## 3. Results

### 3.1. Depletion of Membrane Cholesterol Produces Profound Effects on SERT Function

**Figure 1:**
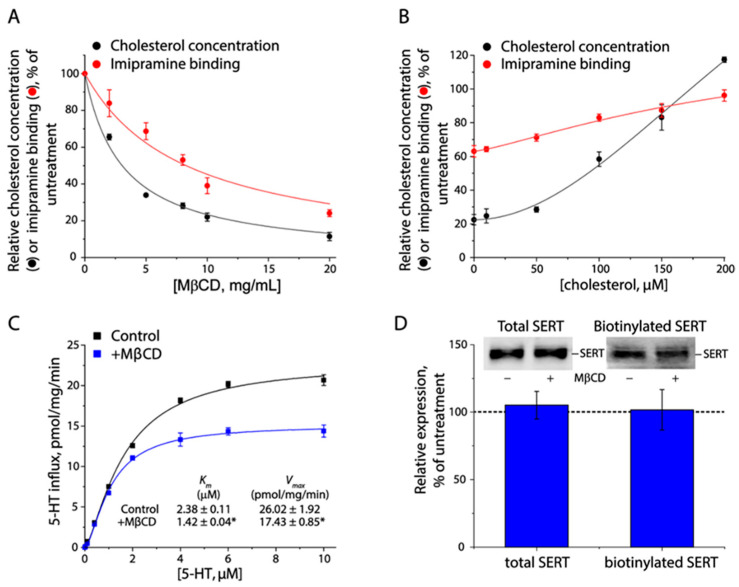
Effects of membrane cholesterol on SERT function. (**A**) Effect of cholesterol depletion on imipramine binding. Crude membrane fractions isolated from HEK 293T cells stably expressing SERT were incubated with MβCD at the indicated concentrations and imipramine binding was then measured. Residual cholesterol was measured in parallel using a cholesterol assay kit. (**B**) Effect of cholesterol replenishment on imipramine binding. Crude membranes were pre-treated with 8 mg/mL MβCD for 30 min and then incubated with a MβCD-cholesterol mixture at the indicated concentrations for an additional 2 h. After washing, membranes were used for measuring imipramine binding and cholesterol concentrations. (**C**) Effect of cholesterol depletion on kinetics for 5–HT uptake. Cells pre-treated with or without 8 mg/mL MβCD were incubated with 5–HT at various concentrations generated by adding unlabeled 5–HT to a constant concentration (20 nM) of [^3^H]5–HT and 5–HT accumulated in the cells was then measured. * *p* < 0.05, compared to those measured without cholesterol depletion (*n* ≥ 3). (**D**) Effects of cholesterol depletion on SERT expression. Cells pre-treated with or without 8 mg/mL MβCD were used for examining SERT expression in total cell lysates (upper left) or on the cell surface by biotinylation (upper right) (*n* = 3). The original immunoblots are shown in Figure S2. A dotted line represents SERT expression in the control cells without MβCD treatment.

To understand the role of cholesterol in SERT function, we systematically examined the effects of cholesterol contents in the plasma membrane on inhibitor binding, transport kinetics, and cell surface expression of SERT with a HEK 293 cell line stably expressing C-terminal FLAG-tagged hSERT. Imipramine binding by SERT was proportionally impaired as cholesterol contents in the plasma membrane were gradually decreased by treating the cells with a cholesterol-chelating agent MβCD at various concentrations (Figure 1A). In addition, SERT binding capability for imipramine was recovered by cholesterol replenishment through incubation of MβCD-pretreated cells with cholesterol–MβCD complexes (Figure 1B). We then investigated the effect of membrane cholesterol on 5–HT transport by SERT. As shown in Figure 1C, cholesterol depletion significantly reduced both *V_max_* for 5–HT transport and *K_m_* for 5–HT. Moreover, immunoblot and biotinylation analyses showed that cholesterol depletion did not alter the total or cell surface expression of SERT (Figure 1D). Our further study with an additional HeLa stable cell line showed that cholesterol depletion produced similar effects on SERT binding and transport activities to those observed with HEK 293T cells (Figure S1), indicating that the regulation of SERT by cholesterol is a ubiquitous event. These results have suggested that cholesterol plays a critical role in the regulation of SERT function, possibly through its direct interaction with SERT rather than enhancement of SERT stability in the plasma membrane.

### 3.2. The Proposed Two Intramolecular Cholesterol Occupation Sites in hSERT

**Figure 2:**
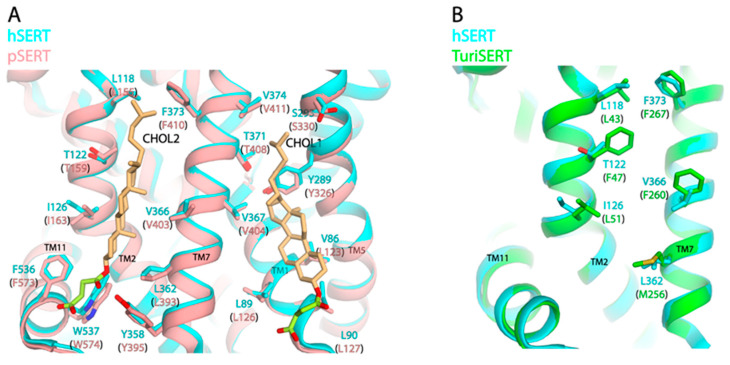
Structural superimposition. (**A**) Superimposition of hSERT (PDB, 7LIA, outward-open) on pSERT (8DE4, outward-open). Two CHS molecules bound at the CHOL1 and CHOL2 sites in pSERT are shown with hemisuccinate groups colored in green and cholesteryl moieties in light brown. Residues that are proposed to participate in CHS interactions in both hSERT and pSERT are shown. (**B**) Superimposition of hSERT (7LIA, outward-open) on a TuriSERT structural model [35]. Residues in the proposed CHOL2 site in hSERT and the corresponding residues in TuriSERT are shown.

Figure 2A shows a structural superimposition of hSERT on pSERT bound with two CHS molecules at the two cholesterol sites (CHOL1 and CHOL2). In hSERT, the CHOL1 site was formed by the residues from TM1, 5, and 7 [9], whereas the CHOL2 site was proposed to be formed by the side chains of Leu118, Thr122, and Ile126 in TM2; Tyr358, Leu362, Val366, and Phe373 in TM7; and Phe536 and Trp537 in TM11, based on the structural alignment of hSERT with pSERT. Because Tyr395 (Tyr358 in hSERT), Phe536 (Phe573), and Trp537 (Trp574) were demonstrated to participate in the interactions with hemisuccinate group of CHS in pSERT [28], we therefore focused on the residues that interact with cholesteryl moiety for further analysis. In addition, we also superimposed the proposed CHOL2 site of hSERT on the model of a bacterial orthologue, TuriSERT, which had recently been identified from a gut bacterium, *Turicibacter sanguinis* [35]. Strikingly, two residues, Thr122 and Val366, in the CHOL2 site of hSERT are displaced by two bulky residues, Phe47 and Phe260, at the equivalent positions in TuriSERT (Figure 2B). Due to the lack of cholesterol in the bacterial membranes, we would assume that the two phenylalanine residues might play a role in the regulation of TuriSERT function similar to that of a cholesterol molecule at the CHOL2 in hSERT.

### 3.3. Mutagenesis Analysis for the CHOL2 Residues in hSERT

**Figure 3:**
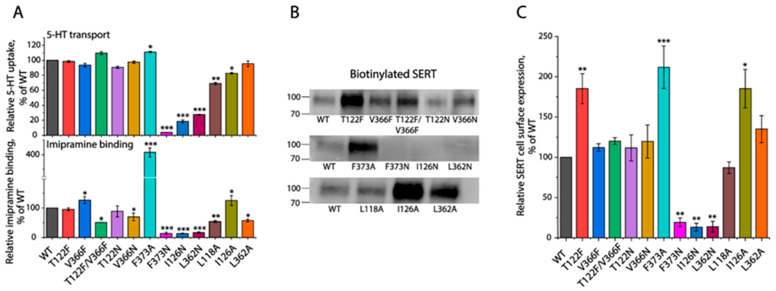
Effects of CHOL2 mutations on 5–HT transport, imipramine binding, and cell surface expression. (**A**) Transport and imipramine binding activities of CHOL2 mutants. Cells transfected with plasmids encoding SERT–WT or CHOL2 mutants were used for measuring 5–HT transport by incubating with 20 nM [^3^H]5–HT (upper) or for imipramine binding by incubating crude membranes with 2 nM [^3^H]imipramine (lower). (**B**) Immunoblot analysis for biotinylated SERT. Cells expressing SERT–WT or its mutants were biotinylated, and biotinylated SERT was then analyzed by immunoblot. The original immunoblots are shown in Figure S2. (**C**) Quantification of biotinylated SERT on the cell surface. * *p* < 0.05, ** *p* < 0.01, *** *p* < 0.001, compared to SERT–WT (*n* ≥ 3).

To illustrate the role of cholesterol at the CHOL2 site in the regulation of SERT, we examined the effects of CHOL2 mutations on transport activity, inhibitor binding, and cell surface expression of SERT. A transport assay performed with 20 nM of [^3^H]5–HT indicated that most of the mutants exhibited comparable abilities for the 5–HT uptake with WT except for three—F373N, I126N, and L362N—which had initial transport activities below 30% of WT (Figure 3A, upper). In addition, our imipramine binding assay indicated that eight mutants—T122F, V366F, T122F/V366F, T122N, V366N, L118A, I126A, and L362A—exhibited 50–130% binding capabilities of WT, while three others—F373N, I126N, and L362N—showed extremely low binding activities. Notably, the F373A mutant exhibited a four-fold increase in imipramine binding, compared to WT (Figure 3A, lower). We then performed biotinylation experiments to examine the effects of CHOL2 mutations on SERT cell surface expression. Our data indicated that the cell surface expression levels of three mutants—T122F, F373A, and I126A—were significantly higher, whereas the other three—F373N, I126N, and L362N—were dramatically lower than WT (Figure 3B,C). By comparison, the rest of the mutants showed comparable cell surface expression with WT. Taken together, extremely low 5–HT transport and imipramine binding activities of the three mutants—F373N, I126N, and L362N—were apparently due to their low cell surface expression. However, with an increased cell surface expression, F373A exhibited a tremendous increase in imipramine binding but not its transport activity. In addition, the T122F and I126A mutants did not translate their enhanced cell surface expression into transport and imipramine binding capabilities.

Next, we performed transport analyses with a range of 5–HT concentrations to investigate the effects of CHOL2 mutations on the transport kinetics for 5–HT uptake (Table 1). Compared to WT, three mutants—T122F, V366F, and T122F/V366F—showed comparable *K_m_* values for 5–HT, whereas six other mutants—T122N, V366N, F373A, L118A, I126A, and L362A—exhibited significantly decreased *K_m_* values. In addition, the *V_max_* values were altered in all CHOL2 mutants; it was decreased in two mutants, V366N and L118A, while increased in seven others. After normalizing its cell surface expression, the specific activity (*V_max_*/surface expression) was shown to be significantly reduced in five mutants, namely T122F, V366N, F373A, L118A, and I126A. By contrast, four other mutants—V366F, T122F/V366F, T122N, and L362A—showed a specific activity comparable with WT. It should be emphasized that four of the five mutants with reduced specific activities also showed significantly lower *K_m_* values than WT.

### 3.4. CHOL2 Mutations Impair MβCD-Induced Inhibition of Imipramine Binding

**Figure 4:**
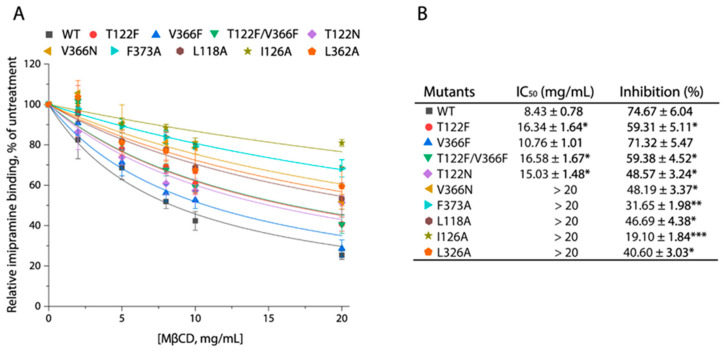
Effects of CHOL2 mutations on MβCD-induced inhibition of imipramine binding. Crude membrane fractions were prepared from the cells expressing SERT–WT or mutants and used for examining MβCD-induced inhibition of imipramine binding as described under Section 2. (**A**) Representative MβCD inhibition curves of imipramine binding with SERT–WT and its CHOL2 mutants. (**B**) MβCD IC_50_ values estimated from the inhibition curves and inhibition of imipramine binding by MβCD at 20 mg/mL. Data represent the mean ± SEM (*n* ≥ 3). * *p* < 0.05, ** *p* < 0.01, *** *p* < 0.0001, compared to WT.

To see if CHOL2 mutations influenced cholesterol occupation, we examined the effects of CHOL2 mutations on MβCD-induced inhibition of imipramine binding. Imipramine binding activity of SERT–WT was remarkably inhibited by MβCD in a concentration-dependent manner, with an estimated IC_50_ value of 8.74 ± 0.78 mg/mL and 74.67 ± 6.04% inhibition at the maximal concentration of MβCD used (20 mg/mL) (Figure 4A,B). By comparison, most of the CHOL2 mutations significantly impaired MβCD-induced inhibition of imipramine binding, leading to increased MβCD IC_50_ values, except for one mutation, V366F, which showed a MβCD IC_50_ value comparable to WT. Specifically, substitutions of Leu118, Ile126, Leu326, or Phe373 by alanine and Val366 by asparagine profoundly reduced MβCD-induced inhibition of imipramine binding with IC_50_ values of more than 20 mg/mL. In addition, the replacement of Thr122 by phenylalanine decreased MβCD inhibition, leading to an approximate two-fold increase in MβCD IC_50_. The effect of a double mutation, T122F/V366F, on MβCD-induced inhibition of imipramine binding was apparently attributable to an imposition of the T122F rather than the V366F mutation. Taken together, these results have suggested that almost all CHOL2 mutations significantly decreased cholesterol occupation at the CHOL2 site, thereby leading to remarkable increases in MβCD IC_50_ values, which was consistent with the proposal that a cholesterol molecule exists at the CHOL2 site in hSERT.

### 3.5. CHOL2 Mutations Stabilize an Inward-Facing Conformation of SERT

**Figure 5:**
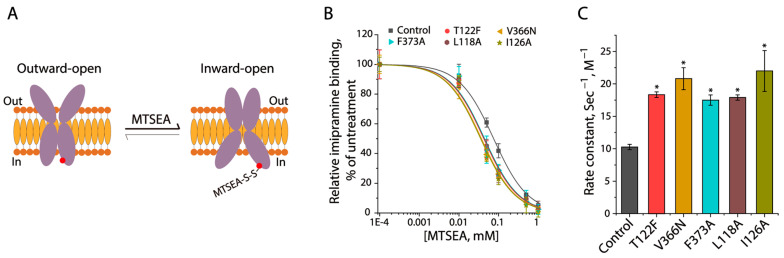
CHOL2 mutations stabilization of an inward-facing conformation of SERT. (**A**) Schematic presentation of MTSEA modification of a cytoplasmic cysteine. A red solid circle represents the Cys277 in the cytoplasmic pathway. (**B**) MTSEA inhibition of imipramine binding. Crude membranes of the cells expressing S277C (control); T122F/S277C; V366N/S277C; F373A/S277C; L118A/S277C; or I126A/S277C were used for examining the inhibition of imipramine binding by MTSEA at a range of concentrations (0–1 mM). (**C**) Rate constants of S277C and the CHOL2 mutants in the S277C background for their reactivities with MTSEA. The MTSEA IC_50_ for S277C or each of the indicated CHOL2 mutants was converted to a pseudo-first-order rate constant of reactivity for Cys277 with MTSEA. A mutant with a higher rate constant represents its faster reactivity with MTSEA, reflecting a more inward-facing conformation than its background S277C. * *p* < 0.05, compared to the background construct S277C (*n* ≥ 3).

To explore the conformational role of a cholesterol molecule at the CHOL2 site, we examined the effects of CHOL2 mutations on SERT conformation. To this end, we employed an approach to evaluate SERT conformation in the cytoplasmic permeation pathway by measuring the effects of CHOL2 mutations on cysteine accessibility [36]. The cysteine mutant used for this study was S277C in a cysteine-less background X5C, in which a cysteine residue was strategically positioned in the cytoplasmic pathway [37]. Several CHOL2 mutations that had resulted in significantly decreased specific activities were introduced into the control construct S277C/X5C, and the resulting cysteine mutants were employed to examine their accessibility to MTSEA under the basal ionic conditions (only NMDGCl in Hepes buffer) in the membrane preparations. SERT is believed to be present in a dynamic equilibrium between the outward-facing and inward-facing states (Figure 5A). The introduction of a bulky group in the cytoplasmic pathway by MTSEA modification of Cys277 limited the cytoplasmic pathway closing and the extracellular pathway opening, thereby resulting in an inhibition of imipramine binding. As shown in Figure 5B, imipramine binding by the control construct S277C/X5C was significantly inhibited by MTSEA in a concentration-dependent manner. The MTSEA concentration leading to a half-maximal inhibition was determined and converted to a pseudo-first-order rate constant of reactivity for Cys277 with MTSEA, which was used to assess its accessibility (Figure 5C). Compared to the control, all CHOL2 mutants tested showed their inhibition curves shifting to lower MTSEA concentration ranges, indicating that the CHOL2 mutations led to higher rate constants of reactivity with MTSEA. These results indicated that the substitutions of these CHOL2 residues stabilized SERT in a more inward-facing conformation.

### 3.6. Cholesterol Mediates the Protomer–Protomer Interactions

**Figure 6:**
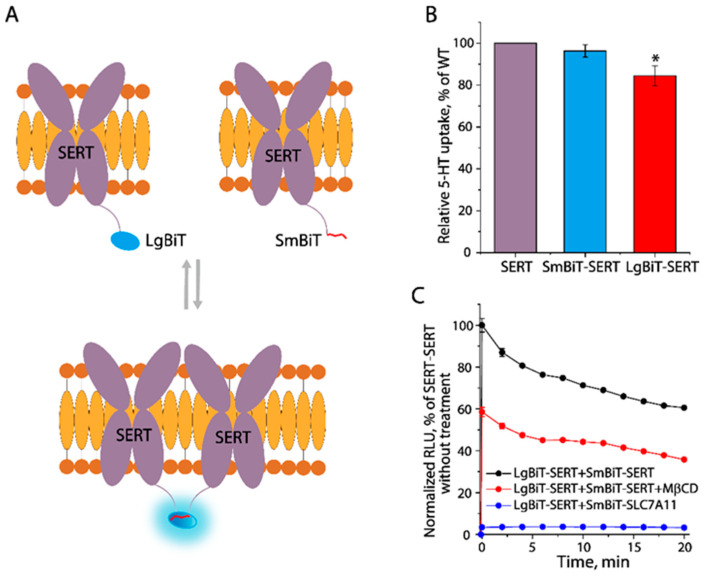
Cholesterol mediation of the protomer–protomer interactions. (**A**) Schematic presentation of the NanoBiT luciferase binary approach for examining the protomer–protomer interactions. Large (LgBiT) or small (SmBiT) subunits were fused to the N-terminus of SERT. (**B**) Transport assay of cells transfected with a plasmid encoding SERT, SmBiT–, or LgBiT–SERT by incubating with 20 nM [^3^H]5–HT. The uptake of 5–HT was expressed as a percentage of the transport activity with the cells expressing SERT. * *p* < 0.05 (*n* ≥ 3), compared to SERT. (**C**) Time course of the luminescent signals produced by the NanoBiT luciferase activity. The luminescent signals were normalized to a maximal signal generated by the cells co-expressing LgBiT–SERT and SmBiT–SERT. RLU, relative luminescence units.

To evaluate cholesterol’s role in SERT dimerization, we employed a NanoBiT luciferase binary approach based on its two-subunit structure for intracellular detection of protomer–protomer interactions in SERT dimerization [38,39]. Either the large (LgBiT) or small (SmBiT) subunit was fused to the N-terminus of SERT. When co-expressed, the interactions between LgBiT– and SmBiT–SERT monomers could be detected when measuring luminescent signals generated by close proximity of luciferase subunits upon the addition of its cell-permeable substrate furimazine (Figure 6A). We first performed the transport assay to examine their capabilities of the NanoBiT-fused SERT for the uptake of 5–HT. As shown in Figure 6B, compared to the control SERT construct, SERT fused with LgBiT or SmBiT showed a comparable 5–HT transport activity, indicating the fusion of a subunit of luciferase to the N-terminus of SERT had a limited effect on SERT function. We then co-expressed LgBiT–SERT with SmBiT–SERT or an unrelated SmBiT–cysteine/glutamate exchanger SLC7A11 and examined the time course of luminescent signals generated by the luciferase activity. The luminescent signals rapidly reached a maximal value once the substrate was added and then decayed slowly with the accompanying substrate consumption (Figure 6C). Compared to the control (LgBiT–SERT + SmBiT–SLC7A11), cells co-expressing LgBiT–SERT and SmBiT–SERT yielded remarkedly strong luminescent signals, indicating LgBiT– and SmBiT–SERTs had specifically interacted in the cells. Strikingly, a treatment with 8 mg/mL of MβCD significantly reduced though not completely inhibited luminescent signals, suggesting the protomer-protomer interactions in SERT dimerization was, at least partially, mediated by cholesterol.

### 3.7. A Potential Cholesterol Site in the Dimeric Interface

**Figure 7:**
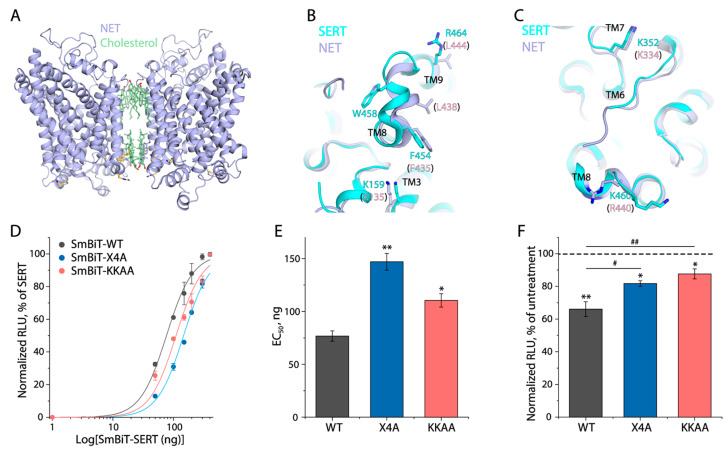
A cholesterol site in the dimeric interface. (**A**) The cholesterol-mediated dimeric structure of NET with each protomer in an outward-open conformation (PDB, 8YR2). (**B**) Superimposition of hNET (8YR2, outward-open) on hSERT (7LIA, outward-open). A set of four residues (Lys135, Phe435, Leu438, and Leu444) forming a cholesterol site in the hNET dimeric interface and the corresponding residues in hSERT are shown. (**C**) Superimposition of hNET (8YR2, outward-open) on hSERT (7LIA, outward-open). Two lysine residues participating in PIP2 binding in hSERT and the equivalent residues in hNET are shown. (**D**) NanoBiT curves of SERT–WT and its mutants, X4A, and KKAA. Luminescent signals are normalized to the maximal value generated by WT. (**E**) The proximity between protomers of SERT–WT and its mutants. EC_50_ is defined as the quantity of SmBiT–SERT required to achieve 50% of the maximal effect in the NanoBiT assay. * *p* < 0.05, ** *p* < 0.01, compared to WT (*n* ≥ 3). (**F**) Effect of MβCD on the proximity between protomers of WT and mutants. NanoBiT luminescent signals of WT and its mutants were measured with the cells pre-treated with or without 8 mg/mL MβCD and normalized to the values obtained from the cells without MβCD treatment (dotted line). * *p* < 0.05, ** *p* < 0.01, compared to the control without MβCD treatment. ^#^ *p* < 0.05, ^##^ *p* < 0.01, compared to WT (*n* ≥ 3).

Recently resolved structures of NET showed a unique dimer interface that was mediated mainly by cholesterol and lipid molecules (Figure 7A). Further structural and functional analyses for NET identified a set of four residues consisting of Lys135 (Lys159 in SERT), Phe435 (Phe454), Leu438 (Trp458) and Leu444 (Arg464) that can play a critical role in cholesterol-mediated NET dimerization [27]. In addition, two positively charged residues, Lys352 and Lys460, were previously shown to directly bind phosphatidylinositol 4,5–bisphosphate (PIP2), yielding a significant effect on the oligomeric distribution of SERT [40,41,42]. As indicated by Figure 7B,C, there are structural alignments between the residues that participate in cholesterol or PIP2-mediated dimerization or oligomerization in NET and the corresponding residues in SERT. To see if SERT had a similar mode of dimerization to NET, we constructed a SERT quadruple mutant (X4A, K159A/F454A/W458A/R464A) and examined its NanoBiT signals with the KKAA mutant (K352A/K460A) as a control. NanoBiT signals were obtained from the cells transfected with a constant amount of LgBiT–SERT cDNA while gradually increasing amount of cDNA encoding SmBiT–SERT, and luminescence curves were then plotted as a function of the SmBiT–SERT cDNA amounts (Figure 7D). The EC_50_ value of the SmBiT–SERT cDNA amount leading to a half-maximal luminescent signal was used to evaluate the proximity between SERT protomers (Figure 7E). SmBiT–KKAA showed a considerably increased EC_50_ value compared to WT. In addition, SmBiT–X4A also showed a remarkably increased EC_50_, indicating that the quadruple substitutions in X4A substantially reduced the protomer–protomer interactions. Moreover, we further examined the effects of MβCD treatment on the NanoBiT signals of these mutants. As shown in Figure 7F, although MβCD treatment decreased the NanoBiT signals of the X4A and KKAA mutants, the signal reductions in either mutant were significantly smaller than WT, suggesting that there were possibly additional cholesterol sites at the dimeric interface. Nevertheless, these results indicated that mutations of the four residues decreased cholesterol-mediated interactions between protomers, supporting the presence of a cholesterol site formed by these residues in the dimeric interface of SERT.

### 3.8. Functional Consequence of Disrupting the Potential Cholesterol Site in the Dimeric Interface

**Figure 8:**
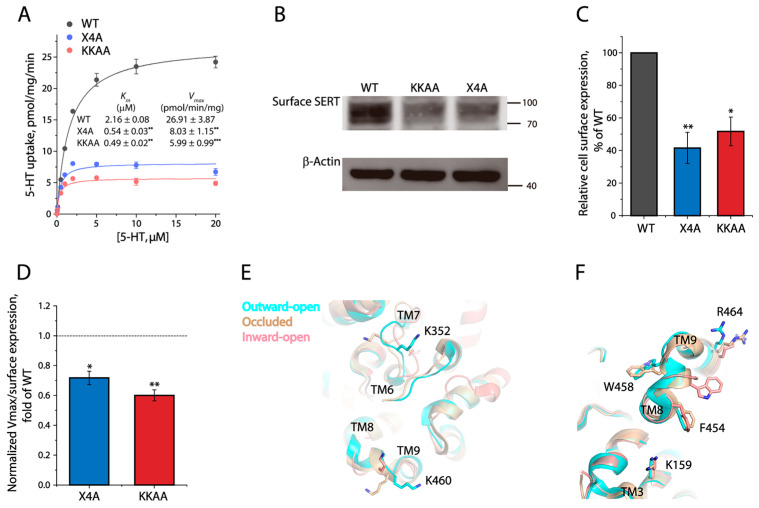
Functional effects of the mutations on a potential cholesterol site at the dimeric interface of SERT. (**A**) Kinetic analysis for the dimeric interface mutants. A transport assay was performed by incubating cells expressing SERT–WT or its mutants (X4A or KKAA) with 5–HT at a range of concentrations generated by adding unlabeled 5–HT to a constant concentration of [^3^H]5–HT (20 nM), as described under Section 2. The insert shows the kinetic parameters for SERT–WT, X4A, and KKAA. ** *p* < 0.01, *** *p* < 0.001, compared to WT (*n* ≥ 3). (**B**) Immunoblot analysis for biotinylated SERT–WT, X4A, and KKAA. The original immunoblots are shown in Figure S2. (**C**) Quantification of biotinylated SERT. SERT cell surface expression was expressed as a percentage of SERT–WT immunoreactivity after being normalized to the immunoreactivity of a housekeeping protein β–actin. * *p* < 0.05, ** *p* < 0.01, compared to SERT–WT (*n* = 3). (**D**) Relative specific transport activity (*V_max_*/SERT cell surface expression) of X4A and KKAA mutants to SERT–WT. A dotted line represents the value of WT. * *p* < 0.05, ** *p* < 0.01, compared to WT (*n* ≥ 3). (**E**,**F**) Structural alignments of hSERT in outward-open (7LIA), occluded (7MGW), and inward-open (7LI9) conformations, as shown by (**E**) two lysines participating in PIP2-mediated dimerization, and (**F**) a set of four residues participating in cholesterol-mediated dimerization.

To investigate the functional role of cholesterol-mediated interactions between SERT protomers, we carried out kinetic analysis for 5–HT uptake (Figure 8A). The KKAA mutant showed a dramatically decreased 5–HT uptake with a *V_max_* value of approximately 22% of WT, while its *K_m_* was more than four times less than WT. Interestingly, the X4A mutant behaved similarly to KKAA in 5–HT transport; its *V_max_* and *K_m_* values were significantly decreased by approximately three- and four-fold, respectively. We then performed biotinylation experiments to determine the expression levels of the mutants on the cell surface. As shown in Figure 8B,C, the cell surface expression levels of X4A or KKAA were approximately 40% and 50% of WT, respectively, indicating that these mutations also led to a significant reduction in their cell surface expression. Thus, the specific transport activity (*V_max_* was normalized to the cell surface expression) of X4A and KKAA was estimated to be approximately 72% and 60% of WT, respectively (Figure 8D), suggesting that these mutations of a cholesterol or PIP2 site in the dimeric interface significantly impaired the specific transport activity with a decreased *K_m_* value.

## 4. Discussion

The present study indicated that MβCD depletion of membrane cholesterol resulted in decreases in both *V_max_* for 5–HT transport and *K_m_* for substrate 5–HT, which was consistent with the previous observations with SERT and other MAT members [19,22,40]. Importantly, our biotinylation experiments showed that MβCD depletion did not alter SERT cell surface expression. Because cholesterol molecules were positioned away from the central cavity for substrate or ion binding in the structures of MATs, we have therefore proposed that such a reduction in SERT catalytic activity was possibly attributable to a cholesterol depletion-induced conformational restriction that slowed down the transport process.

Our mutagenesis study examined cholesterol occupation at a conserved cholesterol site, CHOL2, in hSERT and its functional consequence. Our results showed that mutations of the CHOL2 residues remarkably reduced the MβCD-induced decrease in ligand binding, consistent with an impairment of cholesterol occupation in the CHOL2 mutants. Our accessibility measurements indicated that the CHOL2 mutations induced SERT in an inward-facing conformation, supporting a conformational role of cholesterol occupation at the CHOL2 site in the regulation of SERT. The transport cycle of SERT is a complicated process, which includes 5–HT binding to SERT in an outward-facing conformation, translocation of SERT to an inward-facing conformation by a conformation conversion, 5–HT release into the cytoplasm, and a return to an outward-facing conformation of an empty transporter by an additional conformation conversion [7]. The empty transporter return has been demonstrated to be a rate-limiting step in the transport cycle, which can suppress *K_m_* to a value below the substrate binding affinity for the carrier [43,44]. Previous studies of DAT kinetics have indicated that *K_m_* can become smaller with a slower return step but higher with a faster return step [22,44]. In addition, the motions of the bundle domain comprising TMs 1, 2, 6, and 7, of which TMs 2 and 7 are major components of the CHOL2 site in SERT switching conformations between an outward-facing and an inward-facing state during the transport process, are important for effective transport [26,45]. Interactions that include the bundle domain need to be overcome for conformational conversions. Thus, we have assumed that stabilizing an inward-facing conformation by reducing cholesterol occupation at the CHOL2 site should increase the interactions that need additional energy for breaking to complete the return step of an empty transporter in the transport cycle, thereby leading to deceleration of 5–HT transport with both decreased *V_max_* and *K_m_* values. On the other hand, cholesterol occupation at the CHOL2 site could stimulate the return step by modulating SERT conformation toward an outward-facing state. Interestingly, the conformational role of a cholesterol molecule at the CHOL2 was similar to that at the CHOL1 site, as reported previously in reference [25]. Thus, we propose that two cholesterol molecules at the CHOL1 and CHOL2 sites work cooperatively to promote the conformation conversion of SERT to an outward-facing state in the transport cycle.

Our NanoBiT assay demonstrated that cholesterol mediated the interactions between SERT protomers, although all structures of SERT were in a monomeric form. Earlier biochemical experiments using co-immunoprecipitation, cross-linking, or Förster resonance energy transfer approaches have demonstrated that SERT formed dimers in the plasma membrane [42,46,47,48]. The first structure in a dimeric state with TMs 9 and 12 in the interface was resolved with the leucine transporter (LeuT), a bacterial member of the NSS family [49]. Later, the structures of SERT and DAT showed a pronounced kink in the middle of TM12, suggesting that they formed dimers by a mechanism different from that of LeuT [10,11,50,51]. Notably, the structures of NET were recently determined as a unique cholesterol and lipid-mediated dimer, in which cholesterol molecules were packed against TMs 3, 4, 9, and 12 in each protomer [27]. The mutagenesis study of NET indicated that the mutations of a set of four residues in the dimeric interface led to a remarkable reduction in NET dimerization [27]. Our results from the NanoBiT assay for the X4A mutant showed that a substitution of the equivalent residues also significantly impaired the interactions between the SERT protomers, suggesting SERT, at least partially, shared a similar dimerization mode to NET. However, the fact that the NanoBiT signals of the X4A mutant were still responsive to the MβCD depletion of cholesterol suggested the presence of additional cholesterol sites, as seen in the structures of NET [27]. It was also reasoned that these residues had only 50% identity and that the side chain of W458 stretched out in an opposite direction in SERT compared to that of the corresponding residue L438 in NET, which could result in the difference in cholesterol binding modes between SERT and NET (Figure 7B). In addition to the cholesterol-mediated dimerization of SERT proposed here, previous biochemical experiments have demonstrated that an endogenous cysteine residue C306, located at the end of the third extracellular loop connecting TMs 5 and 6, was involved in the interfacial interactions in DAT dimerization [48,52]. The corresponding residue in other NSS members, such as SERT, NET, and the glycine transporter, was also identified by cross-linking experiments, playing a key role in their dimerization [48,53]. Based on these observations, TMs 6, 11, and 12 have been proposed to constitute a putative dimer interface [47,51,54]. Taken together, these studies have suggested that it is possible for the MATs to dimerize through different interfaces. Therefore, the structural determination of the NSS transporters in various dimeric states is required for our understanding of their flexibility in dimeric assembly.

PIP2-mediated oligomerization of SERT has been demonstrated to be crucial for amphetamine-induced substrate efflux but not for substrate uptake [41]. Moreover, two positively charged residues (K352 and K460) were identified participating in PIP2 binding to SERT. The mutation of both residues to alanine led to a significant alteration of oligomer distribution with a predominant increase in monomers by disrupting PIP2 binding, thus yielding a loss of an amphetamine-induced SERT-mediated efflux and current [41,42]. However, the effect of these lysine residues on 5–HT transport has been disputed. A mGFP-tagged KKAA double mutant showed similar *V_max_* and *K_m_* values as WT in an early study, in which a neurotoxin, methyl–4–phenylpyridinium (MPP^+^), rather than its real substrate 5–HT was used for examining transport kinetics [41]. By contrast, a recent study indicated that the KKAA mutation led to a dramatic reduction in both *V_max_* and *K_m_* for 5–HT uptake [40]. In agreement with this recent observation, our results indicated that the specific transport activity (*V_max_*/cell surface expression) and *K_m_* value of the KKAA mutant were significantly lower than WT. Thus, we propose that the impaired transport velocity of the KKAA mutant was attributable to a conformational constraint, possibly generated by a loss of PIP2 binding, that slowed down conformational conversion during substrate transport. Our structural alignment of hSERT in several states showed that both lysine residues were conformationally sensitive from an outward-open state, through an occluded state, to an inward-open state (Figure 8E), supporting the conformational role of the lysine residues through PIP2-mediated oligomerization.

Similarly, a quadruple mutation of the potential cholesterol site at the dimeric interface (X4A) also remarkedly decreased both the specific transport activity and *K_m_* value for 5–HT. Like the KKAA mutant, a lower transport rate of X4A was apparently due to its decreased *K_m_* resulting from the effect of the X4A mutation on SERT conformation. Although the conformations of K159, F454, and R464 were changed to a small extent during conformational conversion, the side chain of W458 stretched out in one direction in an outward-open conformation, but in the opposite direction in an inward-open conformation (Figure 8F), indicating that W458 played a key conformational role in substrate transport. Thus, we have assumed that cholesterol occupation at this site involving W458 stimulated conformational conversion for 5–HT transport, while disruption of cholesterol occupation at this site in the X4A mutant yielded a conformational restriction, slowing down conformational conversion with a decrease in both the specific activity and *K_m_* value. Taken together, we propose that cholesterol occupation at the dimeric interface of SERT could play a vital role not only in its dimerization but also in the regulation of its catalytic function.

There were several limitations in our present study. First, several mutations led to reductions in the SERT cell surface expression and transport activity. Although the effects of mutations on cholesterol occupation and transport function were evaluated using the specific activity (*V_max_* was normalized to its cell surface expression) to minimize the effect the mutations had on SERT stability in the membrane, we cannot exclude the possibility that the mutations disrupted or destabilized the transporter structure. Second, while MβCD was used for cholesterol depletion, it may have also affected other lipid binding and membrane structures, potentially destabilizing the membrane transporter. In the study, a final concentration of MβCD at its IC_50_ value (8 mg/mL) rather than a higher concentration was used for minimizing its non-specific effects. Third, an impairment of cholesterol occupation at either the CHOL2 or dimeric interface site reduced the *K_m_* value for 5–HT. The *K_m_* value for the substrate of a membrane transporter was influenced by multiple events during the transport process, which included the transporter conformation, transport cycle rates, and accessibility to the binding site besides the intrinsic substrate binding affinity. Our accessibility measurements showed that the mutations induced a more inward-facing conformation in SERT, consistent with a slowed conformational conversion from an inward-facing to outward-facing state. Although a slower return step in the transport cycle was proposed to be a key factor for the decreased *K_m_*, we cannot rule out the effects of other factors, such as the transport rates in other steps, accessibility to the substrate site, and so on.

The present study uncovered the mechanism underlying cholesterol-mediated regulation of SERT by investigating SERT activity in response to alterations of cholesterol content in the plasma membrane. Statins, the most prescribed medications worldwide, lower cholesterol levels by inhibiting 3-hydroxy-3-methylglutaryl-coenzyme A reductase in the cholesterol biosynthesis [55,56]. By using serotonergic RN46A-B14 cells, simvastatin has been shown to stimulate SERT activity of 5–HT uptake by a cholesterol-independent pathway, possibly through a reduction in lipid isoprenylation [57]. Conversely, animal models have shown an antidepressant-like effect of simvastatin that decreased SERT activity [58] and consequently increased hippocampal 5–HT levels [59]; however, the mechanism of the action of simvastatin on SERT in animal models has not been fully understood. In addition, decreased cholesterol contents have been shown in patients with major depressive disorder [60,61]. Thus, exploring a link between the modulation of SERT function by altered cholesterol levels and the symptoms of psychiatric patients is required for developing novel treatments. Hence, understanding the mechanism by which cholesterol can regulate SERT could have broader implications for the development of therapeutics targeting this important transport protein, such as antidepressants.

## 5. Conclusions

Cholesterol, as an important modulator, plays a crucial role in the regulation of SERT. So far, we know that hSERT contains multiple cholesterol occupation sites including CHOL1, CHOL2, and one at the dimeric interface, of which CHOL2 and the interfacial site are examined in this study. Although the residues forming these sites do not directly participate in 5–HT binding, mutations of these residues do profoundly affect *K_m_* value for 5–HT, suggesting that cholesterol regulates SERT function by a conformational mechanism. Cholesterol molecules at these sites are proposed to promote the conformational conversion from an inward-facing to an outward-facing state, whereas disruption of cholesterol occupation at these sites generates a conformational constraint that slows down the return step, generally resulting in a decreased transport rate and *K_m_*. Thus, we propose that cholesterol binding plays a vital role not only in its dimerization but also in the regulation of its catalytic function.

## Figures and Tables

**Table 1:** Kinetic analysis for the CHOL2 mutants.

SERT	*K_m_* (μM)	*V_max_* (pmol/mg/min)	*V_max_*/Surface Expression
WT	2.38 ± 0.11	26.02 ± 1.92	1
T122F	2.63 ± 0.13	47.58 ± 5.30 **	0.78 ± 0.08 *
V366F	1.72 ± 0.09	42.56 ± 4.33 **	1.07 ± 0.10
T122F/V366F	2.68 ± 0.13	41.08 ± 3.73 **	0.98 ± 0.11
T122N	1.15 ± 0.06 **	36.27 ± 1.69 *	0.99 ± 0.10
V366N	0.90 ± 0.05 **	13.51 ± 1.29 ***	0.35 ± 0.03 **
F373A	1.68 ± 0.08 *	35.82 ± 3.29 *	0.52 ± 0.05 *
L118A	1.53 ± 0.11 *	16.95 ± 1.93 **	0.59 ± 0.04 *
I126A	1.42 ± 0.06 *	58.73 ± 5.66 **	0.83 ± 0.09 *
L362A	1.65 ± 0.04 *	45.52 ± 2.78 **	0.87 ± 0.10

Cells transiently transfected with a plasmid encoding SERT–WT or CHOL2 mutant were used for kinetic analysis by incubating with 5–HT at a range of concentrations generated by adding unlabeled 5–HT to a constant concentration of [^3^H]5–HT. Nonspecific [^3^H]5–HT uptake was determined in the presence of 100 μM fluoxetine. * *p* < 0.05, ** *p* < 0.01, *** *p* < 0.0001 compared to WT (*n* ≥ 3).

## Data Availability

The original contributions presented in this study are included in the article/Supplementary Materials. Further inquiries can be directed to the corresponding author.

## Supplementary Materials

The following supporting information can be downloaded at: https://www.mdpi.com/article/10.3390/biom16030472/s1, Figure S1: Effects of HeLa cell plasma membrane cholesterol on SERT function; Figure S2: The original immunoblotting images in the relevant figures.

## Abbreviations

The following abbreviations are used in this manuscript:

SERT	Serotonin Transporter
5–HT	5–Hydroxytryptamine
CNS	Central Nervous System
SSRIs	Selective Serotonin Reuptake Inhibitors
DAT	Dopamine Transporter
NET	Norepinephrine Transporter
LeuT	Leucine Transporter
NSS	Neurotransmitter Sodium Symporter
MAT	Monoamine Transporter
TM	Transmembrane
MβCD	Methyl–*β*–cyclodextrin
CHS	Cholesteryl Hemisuccinate
MTSEA	2–Aminoethyl Methanethiosulfonate Hydrobromide
KRH	Krebs–Ringer-Hepes
NMDG	1–Deoxy–1–methylamino–*D*–glucitol
MPP^+^	Methyl–4–phenylpyridinium
PBS	Phosphate-Buffered Saline
