# Supplementary material for: The Role of Cholesterol at the CHOL2 Site and the Dimeric Interface in the Regulation of Serotonin Transporter Function and Dimerization
Source: Biomolecules. 2026 Mar 21;16(3):472. doi: 10.3390/biom16030472 (PMC13024735; doi:10.3390/biom16030472)
Supplement: Supplementary file 1 [file biomolecules-16-00472-s001.zip › biomolecules-4195898-supplementary.pdf]

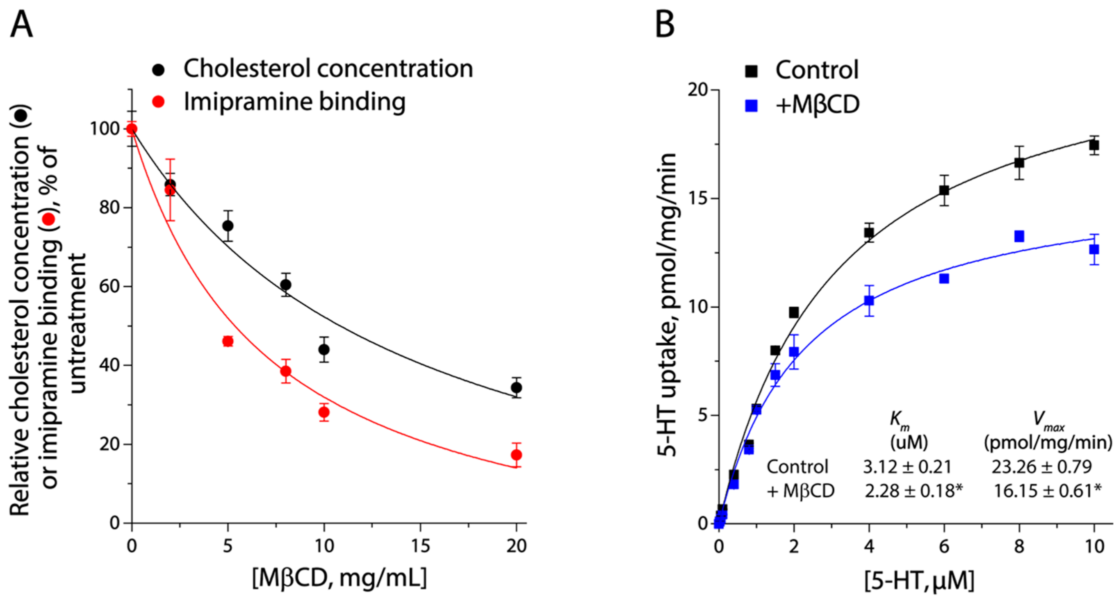

Figure S1. Effects of HeLa cell plasma membrane cholesterol on SERT function. (A) Effect of cholesterol depletion on imipramine binding. Crude membrane fractions isolated from HeLa cells stably expressing SERT were incubated with MβCD at the indicated concentrations and imipramine binding was then measured. Residual cholesterol was also measured in parallel as described under "Materials and Methods". (B) Effect of MβCD depletion of cholesterol on kinetics for 5–HT uptake. HeLa stable cells pre–treated with or without 8 mg/mL MβCD were incubated with 5–HT at various concentrations and 5–HT accumulated in the cells was then measured. *$p < 0.05$, compared to those measured without cholesterol depletion ($n = 3$).

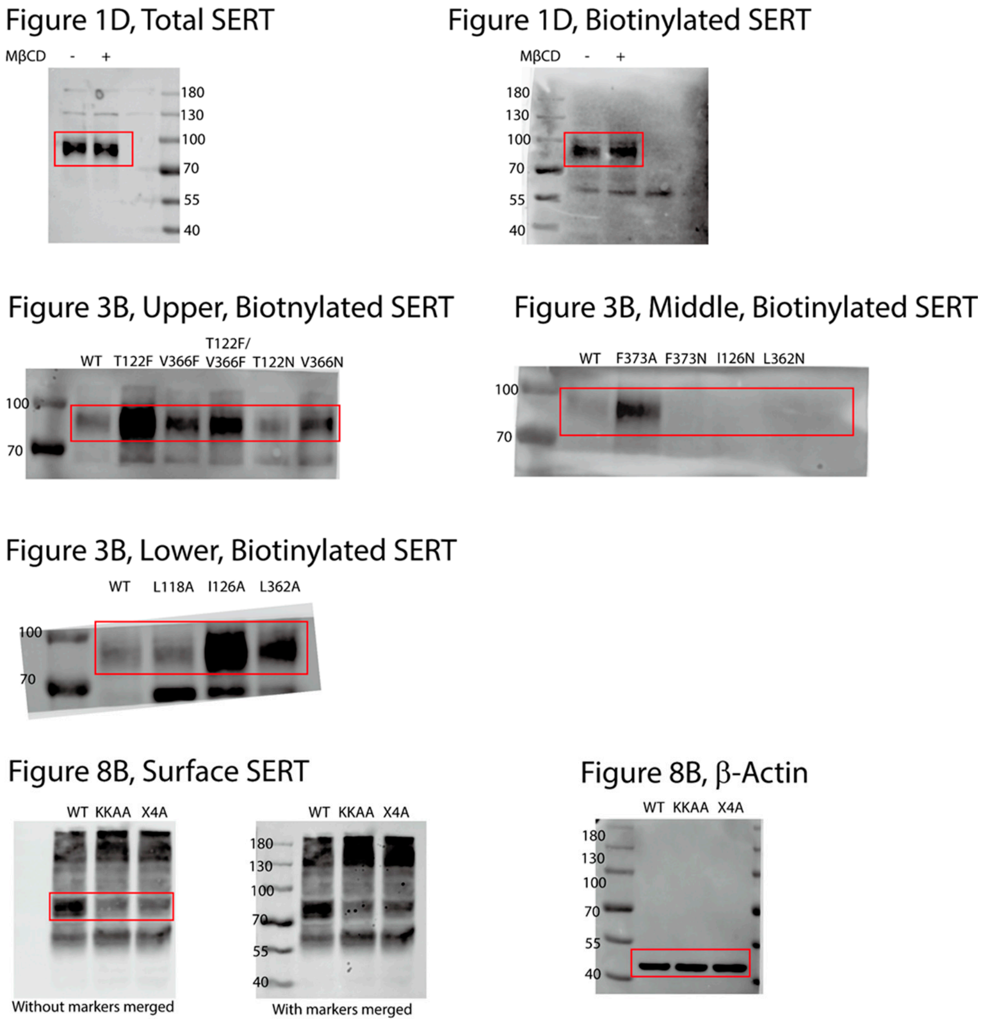

Figure S2. The original immunoblotting images in the relevant figures.
